# Tailored Education Increased Capability and Motivation for Fall Prevention in Older People After Hospitalization

**DOI:** 10.3389/fpubh.2021.683723

**Published:** 2021-08-03

**Authors:** Chiara Naseri, Steven M. McPhail, Meg E. Morris, Terry P. Haines, Christopher Etherton-Beer, Ronald Shorr, Leon Flicker, Max Bulsara, Den-Ching A. Lee, Jacqueline Francis-Coad, Nicholas Waldron, Anne-Marie Hill

**Affiliations:** ^1^Faculty of Health Sciences, Curtin School of Allied Health, Curtin University, Perth, WA, Australia; ^2^Australian Centre for Health Services Innovation and Centre for Healthcare Transformation, Faculty of Health, School of Public Health & Social Work, Queensland University of Technology, Brisbane, QLD, Australia; ^3^Clinical Informatics Directorate, Metro South Health, Brisbane, QLD, Australia; ^4^Healthscope Academic and Research Collaborative in Health, La Trobe University, Bundoora, VIC, Australia; ^5^College of Healthcare Sciences, James Cook University, Douglas, QLD, Australia; ^6^School of Primary and Allied Health Care, Monash University, Frankston, VIC, Australia; ^7^Royal Perth Hospital, Perth, WA, Australia; ^8^Western Australian Centre for Health and Ageing, Medical School, University of Western Australia, Perth, WA, Australia; ^9^Malcom Randall Veterans Affairs Medical Center, Geriatric Research Education and Clinical Center, Gainesville, FL, United States; ^10^College of Medicine, University of Florida, Gainesville, FL, United States; ^11^Institute for Health Research, The University of Notre Dame Australia, Fremantle, WA, Australia; ^12^Department of Geriatric Rehabilitation, Armadale Health Service, Department of Health, Perth, WA, Australia

**Keywords:** fall prevention, hospital discharge, health behavior change, education, post-hospital home falls, older adults, physiotherapy

## Abstract

Recently hospitalized older people are at risk of falls and face barriers to undertaking fall prevention strategies after they return home from hospital. The authors examined the effects of tailored education delivered by physiotherapists on the knowledge (capability) and the motivation of older people to engage in fall prevention after hospital discharge. Utilizing data gathered from a recent trial, data was analyzed from 390 people who were 60 years and over without impaired cognition (>7/10 abbreviated mental test score) and discharged from three Australian hospitals. Motivation and capability were measured at baseline in the hospital and at 6-months after hospital discharge by blinded assistants using structured surveys. Bivariate analysis using generalized linear modeling explored the impact of education on the capability and motivation. Engagement in fall prevention strategies was entered as an independent variable during analysis to determine associations with capability and motivation. The education significantly improved capability [−0.4, 95% CI (−0.7, −0.2), *p* < 0.01] and motivation [−0.8, 95% CI (−1.1, −0.5), *p* < 0.01] compared with social-control at the time of hospital discharge. In contrast, social-control participants gained capability and motivation over the 6-months, and no significant differences were found between groups in capability [0.001, 95% CI (−0.2, 0.2), *p* = 0.9] and motivation [−0.01, 95% CI (−0.3, 0.3), *p* = 0.9] at follow-up. Tailored fall prevention education is recommended around hospital discharge. Participants still needed to overcome barriers to falls prevention engagement post hospitalization. Thus, tailored education along with direct clinical services such as physiotherapy and social supports is warranted for older people to avoid falls and regain function following hospitalization.

## Introduction

The problem of falls and associated ongoing costs for healthcare are recognized to be serious among older people who have recently been discharged home from hospital (1, 2). Apart from having age-related comorbidities (3), this population is recovering from illness or disability, and can experience ongoing effects from being in hospital (4). Heightened falls risk is reflective of discharge care practices and the diminished function of many older individuals (5–7). Underlying effective discharge care is the concept of patient-centered care (8) that values patients' understanding of their own health risks, while helping them to gain necessary health knowledge to maintain safety and independence (9).

Previous studies have shown older people to have limited knowledge about fall prevention following hospitalization (10). Sometimes they believe that fall prevention activities are more important for other older people than for themselves (11). Reduced knowledge about falls risks and low motivation affect the engagement of older people in fall strategies, such as exercise (12, 13). To address this need, we recently implemented a novel education program that was designed based on the COM-B model (14). It aimed to reduce falls in older people by encouraging uptake of fall prevention strategies once they returned home from hospital. However, we did not find any differences in the uptake of fall prevention strategies after returning home from hospital for those who received the education compared with control. It is important to evaluate the constructs of the model that underpinned the education program which may help to explain the lack of uptake of falls prevention behavior in these people. Importantly, it may assist in the refinement of the education program for future use.

The theory of health behavior change proposes that people require the opportunity to make changes within their daily lives, as well as the knowledge and motivation to change their behavior (14). Previous studies of hospital discharge care reported that older people understood discharge plans, yet were unprepared to carry these out when faced with different demands in their living environment (15, 16). Some older people also report difficulty in overcoming barriers (lacked opportunity) to implement fall prevention action plans once they returned home (17). Nonetheless, opportunity is only one of the constructs in the model. The COM-B health behavior change model explains that people may not take-up opportunities or address barriers to undertake fall prevention activities if they lack the capability or motivation (14). Thus, evaluating the capability and motivation of recipients would provide us with a better understanding of the interplay between these constructs, leading to a behavioral change. This study aimed to measure the impact of tailored education on the level of capability (knowledge) and motivation of older people to engage in fall prevention during 6 months after hospitalization, compared with control conditions.

## Methods

### Research Design

A secondary analysis of data from a randomized controlled trial (RCT) (*n* = 390) was published previously (2). This quantitative evaluation used data from three collection points, namely: at baseline in hospital prior to education delivery (T1), following education delivery prior to hospital discharge (T2), and at 6 months after discharge (T3). Ethical approvals were obtained from human research ethics committees of the participating hospitals and universities. All participants provided written informed consent. This study has been reported according to the CONSORT (2010) statement (18).

### Participants and Setting

Participants (*n* = 390) were hospital patients who were aged 60 years and over who were enrolled in a trial and randomized in a trial to receive either a tailored education intervention in addition to the usual care or a social control intervention and followed up after receiving the intervention prior to discharge and at 6-months after discharge from three rehabilitation hospitals in Perth, Western Australia (2). Participants were recovering from a variety of geriatric conditions, including orthopedic, neurological, and general medical conditions. They were included in the trial if they spoke English as a first language, could give written informed consent, and were discharged to the community. Participants were excluded if they were to be discharged to a residential care facility, had hearing or visual problems that excluded them from engaging with the education materials, or had impaired cognition [inclusion criteria >7/10 on abbreviated mental test score (19)]. The protocol for this study has been published (20).

### Capability and Motivation: COM-B Theory of Behavior Change

The behavioral change theory utilized during the design of the education intervention suggests that capability, motivation, and opportunity interact to affect behavioral outcomes (COM-B) (14). For this study, capability and motivation outcomes were framed as internal factors that could be modified by the education, such as their general knowledge about falls risks (capability), self-perceived awareness of their own falls risks (motivation), and willingness to participate in fall prevention strategies (motivation). For example, participants were asked to consider social supports to complete their daily activities when they returned home from hospital, for which they required (capability) knowledge and motivation, as well as the opportunity to access social supports. External components (opportunity) were explored during a separate qualitative study (17) and were considered external social and physical enablers (such as access to therapy and social supports) that may have existed within the environment and life-circumstances of the participants after hospital discharge. This evaluation focused on the internal constructs of capability and motivation from the COM-B model.

### Outcomes

The outcomes for the study were as follows:

- Capability: participant perceived knowledge about the risks of falls and falls injuries.
- Motivation: self-perceived awareness of the participant about their own fall risks, likelihood of reduced independence following hospitalization, and willingness to engage in fall prevention strategies.

Capability and motivation outcomes were measured for both groups by blinded research assistants using structured surveys face to face in hospital prior to allocation at baseline (T1), following the education intervention, but prior to discharge (T2), and by telephone at six months following hospitalization (T3). The surveys were modified from previous studies that evaluated fall prevention behavioral change interventions (10, 21–23), and contained questions that were closed-item statements requiring responses on a five-point Likert response scale, where 1 (“strongly agree”) indicated a better outcome compared to 2 (“agree”), 3 (“undecided”), 4 (“disagree”), and 5 (“strongly disagree”). Survey questions were worded to stimulate a response that would indicate the presence of capability and motivation to engage in fall prevention strategies based on their level of agreement or disagreement. For example, the wording of a survey item pertaining to capability, regarding participant knowledge of falls risks in older people following hospitalization was, “I think that older people who go home from hospital are at risk of falling over in the first 6 months following hospitalization.” The survey was pilot tested on a representative sample of 10 older people recently discharged home from hospital to confirm face, content, and construct validity (24).

### Data Collection and Procedure

Demographic data were collected at baseline (see Table 1) using a structured questionnaire. Prior to discharge, the education group received the education in addition to usual care. The control group received a social intervention in addition to usual care that discussed positive aging without reference to falls prevention. The education was delivered by physiotherapists using a workbook and video (2). It presented fall-prevention strategies specific to the post discharge period and tailored to participants based on their perceived knowledge of falls risks, willingness to participate in falls prevention strategies, and identified barriers to fall prevention engagement after hospitalization. A goal-directed action plan to initiate after hospital discharge was then provided to help prepare participants for their imminent discharge home and it included fall prevention strategies, such as completion of safe exercise, an occupational therapist home hazard assessment, and seeking assistance with daily activities (ADLs) to enable a gradual return of independence (2). The same educators provided guided feedback once per month *via* telephone for 3 months after hospital discharge.

**Table 1:** Demographic characteristics of participants.

**Variable^a^**	**Education**	**Social control**
	***n* = 149**	***n* = 143**
Age, mean (SD)	77.2 (8.9)	77.9 (8.4)
Gender female	90 (60.4)	95 (66.4)
Length of stay in hospital (days): median (IQR)	24 (43–16)	24 (35–18)
Highest education level attained		
Primary	15 (10.1)	23 (16.1)
Grade 10	68 (45.6)	62 (43.4)
Grade 12	17 (11.4)	19 (13.3)
Technical college	27 (18.1)	22 (15.4)
University	22 (14.8)	17 (11.9)
Visual impairment^b^	44 (29.5)	35 (24.5)
Hospital admission in 1 year prior to current	54 (36.2)	67 (46.8)
Fell in 6 months prior to hospital admission	107 (71.8)	99 (69.2)
Fell in hospital prior to discharge	12 (8.0)	12 (8.4)
Discharge destination		
Home alone	64 (42.9)	57 (39.8)
Home with partner	61 (40.9)	54 (37.7)
Home with other	16 (10.7)	29 (20.2)
Other^c^	3 (2.1)	8 (5.3)
Discharge mobility		
No aid	20 (13.4)	24 (16.7)
Walking stick	18 (12.0)	17 (11.8)
Walking frame	96 (64.4)	90 (62.9)
Wheelchair	15 (10.1)	12 (8.2)
Depressed mood, GDS ≥ 5^d^	47 (24)	51 (27)
AQoL^e^ mean (SD)	0.6 (0.1)	0.6(0.1)
ADL Function at discharge		
Katz^f^ median (IQR)	5 (6–3)	5 (6–3)
Lawton's ^g^ median (IRQ)	7 (8–5)	7 (8–6)

a *All data measured in n(%) unless otherwise stated*.

b *Glaucoma, cataracts, macular degeneration*.

c *Transitional Care or Nursing Home*.

d *Geriatric Depression Scale Short Form, score ≥ 5 suggests depression*.

e *AQoL-6D utility instrument*.

f *Katz Index of Independence in Activities of Daily Living, range 0–6 greater score indicates more independence*.

g *Lawton's Instrumental Activities of Daily Living, range 0–8 greater score indicates more independence*.

### Statistical Analysis

This was a *post-hoc* secondary analysis of the outcomes of capability and motivation with consideration of the covariates of engagement in fall prevention strategies. All analyses were conducted using Stata release 16, (StataCorp, College Station, Texas, 2020), the significance level set at = 0.05, and the sample size previously determined by primary trial effect analysis (2). Intention to treat analysis was undertaken to determine influence of group allocation on outcomes based on the trial randomization. Non-parametric Likert scale outcome data were summarized using median and interquartile range (IQR) for both groups at data collection timepoints (T1, T2, T3). Graphs of the proportion of response ratings between 1 and 5 for each outcome at the three timepoints were completed to present the data. Differences in capability and motivation within and between groups, with and without the interaction of time were compared using mixed-effects generalized linear modeling, with adjustment for identified fall risk factors in this population, including older age, previous falls, presence of visual impairment, depressed mood, and use of a walking aid at the time of discharge, consistent with a pilot study of the intervention (21). Further, analysis to determine the association between the presence of capability and motivation (as binary data) and engagement in falls prevention strategies was completed. Data from a previous evaluation (25) regarding the reported engagement of the participants in fall prevention strategies after hospital discharge at 6 months follow-up (T3) was included as an independent variable during analysis, using mixed effects generalized linear modeling with adjustment for falls risk factors. This was completed to identify any association between engagement as behavior change, and the primary outcomes of capability and motivation. The fall prevention strategies were categorized as discrete data and consisted of participants having received assistance with ADLs (such as showering and dressing); instrumental activities of daily living [IADLs, (such as cleaning and shopping)]; completed home (hazard) modifications; and completed an exercise program during the 6 months post hospitalization.

## Results

Participant flow through the study (in Supplementary Figure 1) shows that from the original cohort of 390 participants at baseline, there were 292 who completed measures at 6 months follow-up. There were no significant differences in characteristics between the two groups (Table 1).

Table 2 presents differences in capability and motivation compared between education and control groups at baseline in hospital (T1), follow-up prior to hospital discharge (T2), and 6 months post hospitalization (T3). Participants who received the education significantly improved their capability (knowledge about fall risks of other people and falls injury risks), and motivation (awareness of their own falls risks and loss of independence) compared with control following education delivery in hospital (T2). Education group participants maintained capability and motivation when surveyed at the 6-month follow-up (T3), whereas, those in control gained capability (knowledge) and motivation during their post hospitalization recovery at home, leading to no significant differences between education and control groups at 6-months (T3).

**Table 2:** Difference in capability and motivation compared between education and control groups.

**Outcome item^a^**	**Independent variables^b^**	**Reference variable^b^**	**Coefficient of change^c^**	**95% CI**	***p*-value**
**Capability**					
1. Knowledge of other older people's falls risks following hospitalization	Intervention (overall)	Control (overall)	−0.2	−0.3, −0.1	<0.01^*^
	Intervention at T2	Control at T2	−0.4	−0.7, −0.2	<0.01^*^
	Intervention at T3	Control at T3	0.001	−0.2, 0.2	0.9
2. Knowledge of other older people's falls-injury risks following hospitalization^d^	Intervention	Control	−0.1	−0.2, −0.01	<0.01^*^
	Intervention at T2	Control at T2	−0.4	−0.5, −0.2	<0.01^*^
	Intervention at T3	Control at T3	No data		
**Motivation**					
3. Awareness of own falls risks following hospitalization	Intervention	Control	−0.4	−0.5, −0.2	<0.01^*^
	Intervention at T2	Control at T2	−0.8	−1.1, −0.5	<0.01^*^
	Intervention atT3	Control at T3	−0.01	−0.3, 0.3	0.9
4. Awareness of own risk of falls-injury following hospitalization	Intervention	Control	−0.1	−0.2, 0.02	0.1
	Intervention at T2	Control at T2	−0.7	−0.9, −0.5	<0.01^*^
	Intervention at T3	Control at T3	0.05	−0.2, 0.3	0.7
5. Awareness of own reduced independence following hospitalization	Intervention	Control	−0.4	−0.5, −0.2	<0.01^*^
	Intervention at T2	Control at T2	−1.0	−1.2, −0.7	<0.01^*^
	Intervention at T3	Control at T3	−0.1	−0.4, 0.2	0.4

a *Item is capability or motivation outcome*.

b *Data collection time-variable was introduced at T2: posteducation prior to discharge and T3:6 months post hospitalization using mixed-effects generalized linear modeling*.

c *The coefficient of change: degree of change in outcome where a more negative coefficient indicates a stronger agreement (toward 1) on the Likert scale*.

d *This item was omitted in the final survey*.

* *Significant p-value*.

**Figure 1:**
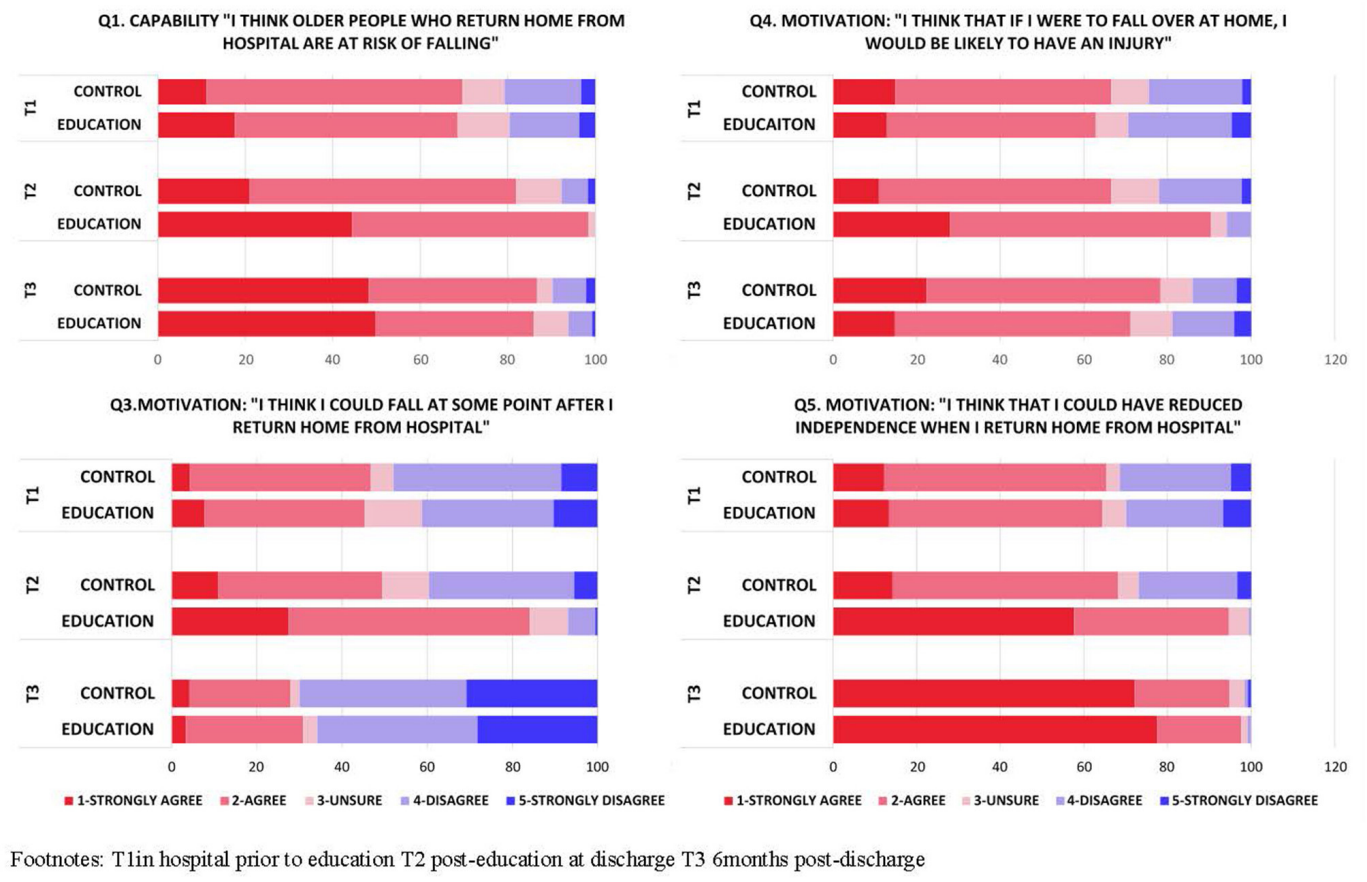
Change in levels of capability and motivation compared between education and control group at three timepoints.

Figure 1 presents participant levels of capability and motivation in both intervention (education) and control groups at baseline (T1), in hospital prior to discharge (T2), and at 6 months post hospitalization (T3). Supplementary Table 1 presents summarized descriptive statistics (median and interquartile range), and Supplementary Table 2 presents complete data (number and percentage) of the levels of capability and motivation for both groups at baseline (T1), at follow-up prior to discharge (T2), and at 6 months post hospitalization (T3).

Significant associations were found between the presence of capability and motivation outcomes and engagement in post hospitalization fall–prevention strategies in both groups (Table 3). Those participants who scored positively (strongly agree and agree) for knowledge (capability) of risk of post hospitalization falls of other older people were significantly more likely to complete an occupational therapist home (hazard) assessment [95% CI, 0.9, 3.1, *p* = 0.05]. Participants in both groups who were more positively aware of their own post hospitalization falls risks (motivation), were significantly more likely to complete home (hazard) modifications [95% CI, 1.0, 2.5, *p* = 0.03]. While participants in both groups who were more positively aware of their own post hospitalization fall-injury risks (motivation) were significantly more likely to ask for formal ADL assistance [95% CI, 1.1, 2.5, *p* < 0.01]. Participants in both groups who were more positively aware of their own risk of losing independence (motivation) were significantly more likely to ask for informal ADL assistance [95% CI, 0.4, 0.9, *p* = 0.02] and to exercise after hospitalization [95% CI, 1.2, 2.6, *p* < 0.01].

**Table 3:** Associations between post hospitalization engagement in fall prevention and presence of capability and motivation within groups.

**Outcome item^a^**	**Fall prevention strategy**	**exp (b)^b^**	**95% CI**	***p*-value**
**Capability**				
Knowledge of other older people's falls risks	OT home assessment	1.7	0.9, 3.1	0.05^*^
**Motivation**				
Awareness of falls risks	Home hazard reduction	1.6	1.0, 2.5	0.03^*^
Awareness of falls-injury risks	Formal ADL assistance	1.7	1.1, 2.5	<0.01^*^
Awareness of reduced independence risks	Exercise post hospitalization	1.8	1.2, 2.6	<0.01^*^
	Informal ADL assistance	0.6	0.4, 0.9	0.02^*^

a *Item is capability or motivation outcome*.

b *Coefficient indicates association of engagement in fall prevention strategy with capability or motivation outcome, where a more positive number shows a greater association*.

* *Significant p-value*.

## Discussion

The key finding of this study was that tailored education delivered in hospital significantly improved the capability and motivation of older people to engage in fall prevention strategies at the time of hospital discharge. Although there were no significant differences between groups at 6-months follow-up (T3), the intervention group maintained their levels of capability and motivation after 6-months post hospitalization.

Tailored fall prevention education in hospital prepares patients for a gradual and safe transition home (26, 27). It also optimizes the capability and motivation of older patients to engage in fall-prevention strategies once home (2, 10). This positive change did not translate into improved or long-lasting engagement in falls prevention strategies post hospitalization (25). This implementation gap was correlated with the finding that fall incidence was not reduced (2). The results support prior studies, which show reduced participation in fall prevention strategies, such as exercises after hospitalization (12, 28). They also support prior trials showing that some older people can be passive when given a list of recommendations (29). Older people often find it easier to complete fall-prevention action plans in the hospital setting, where more structure and support is available, compared with their immediate home environment (30).

Participants who received tailored fall prevention education showed raised motivation and awareness of their own post hospitalization falls risks and likelihood of falls-injury at the time of hospital discharge. This was encouraging given that some older people do not acknowledge their own heightened fall risk (31, 32). Many are reluctant to engage in fall prevention strategies because they do not believe they are at the risk of fall (11, 12, 33).

Education recipients were not only more aware of their own risks of falls (motivation), but they were also more (capable) knowledgeable about the risk of post-discharge falls and falls injuries for other older people, at the time of hospital discharge. In contrast, participants in control group showed raised knowledge only by 6 months follow-up. Those who did not receive the education were more reliant upon their existing health knowledge and experiential learning during the post hospitalization recovery period (34, 35). This is when they are known to be more vulnerable to adverse events such as falls (2, 36).

### Strengths and Limitations

The current study was conducted according to a published protocol and accompanied an RCT (20) that delivered an evidenced-based tailored education intervention with minimal drop-out (2). All outcomes were measured using blinded assessors. Most discharge studies have a limited follow-up period of 30 to 90 days (37, 38), whereas, this study explored the longer-term impact of the education on the capability and motivation for fall-prevention behavior following hospitalization of older people.

Although some external social and environmental demands were considered at the time of education delivery, some were not foreseeable to educators or patients, such as delayed provision of social assistance, and therefore could not be considered during the RCT (2). Educators prepared patients to engage with available supports delivered through hospital and community organizations, however the intervention did not provide direct support in the home and community. Participant experiences of external demands that were faced after hospital discharge, such as the availability and timing of community support, were not explored.

### Conclusion

This study revealed complexities to enable behavioral change in older people who have been recently hospitalized. The tailored education delivered around the time of discharge can be helpful as it improved motivation and capability for fall prevention at the time of discharge. Participants still needed to overcome barriers to implement fall prevention activities once they returned home from hospital. These barriers represent gaps in the living environment and life-circumstances of older people after discharge from hospital. Thus, having some support to overcome these gaps in opportunity after hospitalization appear to be essential steps toward enabling older people to safely regain their independence in their home and community.

## Data Availability Statement

The raw data supporting the conclusions of this article may be made available by the authors upon request.

## Ethics Statement

The studies involving human participants were reviewed and approved by Human Research Ethics Committees of North Metropolitan Health Service and South Metropolitan Health Service with reciprocal approval from the University of Notre Dame Australia and Curtin University. The patients/participants provided their written informed consent to participate in this study.

## Author Contributions

A-MH, CN, SM, and TH conceptualized the current study design and research protocol with ongoing expertise and support from MM, CE-B, RS, LF, MB, D-CL, JF-C, and NW. A-MH and CN led trial management including data collection and management and site procedure, in consultation with TH, MM, CE-B, LF, and NW. A-MH, CN, and SM led statistical analyses with support from TH and MB. CN led the drafting of all sections of the manuscript in consultation with A-MH, SM, MM, D-CL, CE-B, and JF-C. All authors critically revised the manuscript for important intellectual content and read and approved the final version of the manuscript.

## Conflict of Interest

The authors declare that the research was conducted in the absence of any commercial or financial relationships that could be construed as a potential conflict of interest.

## Publisher's Note

All claims expressed in this article are solely those of the authors and do not necessarily represent those of their affiliated organizations, or those of the publisher, the editors and the reviewers. Any product that may be evaluated in this article, or claim that may be made by its manufacturer, is not guaranteed or endorsed by the publisher.

## Supplementary Material

Supplementary Figure 1 Participant flow through the study.

Supplementary Table 1 Descriptive statistics of capability and motivation for education and control groups.

Supplementary Table 2 Capability and motivation: complete Likert-scale responses for education and control groups at baseline and follow-up.
