# Supplementary figures and images for: Tailored Education Increased Capability and Motivation for Fall Prevention in Older People After Hospitalization
Source: Front Public Health. 2021 Aug 3;9:683723. doi: 10.3389/fpubh.2021.683723 (PMC8369365; doi:10.3389/fpubh.2021.683723)

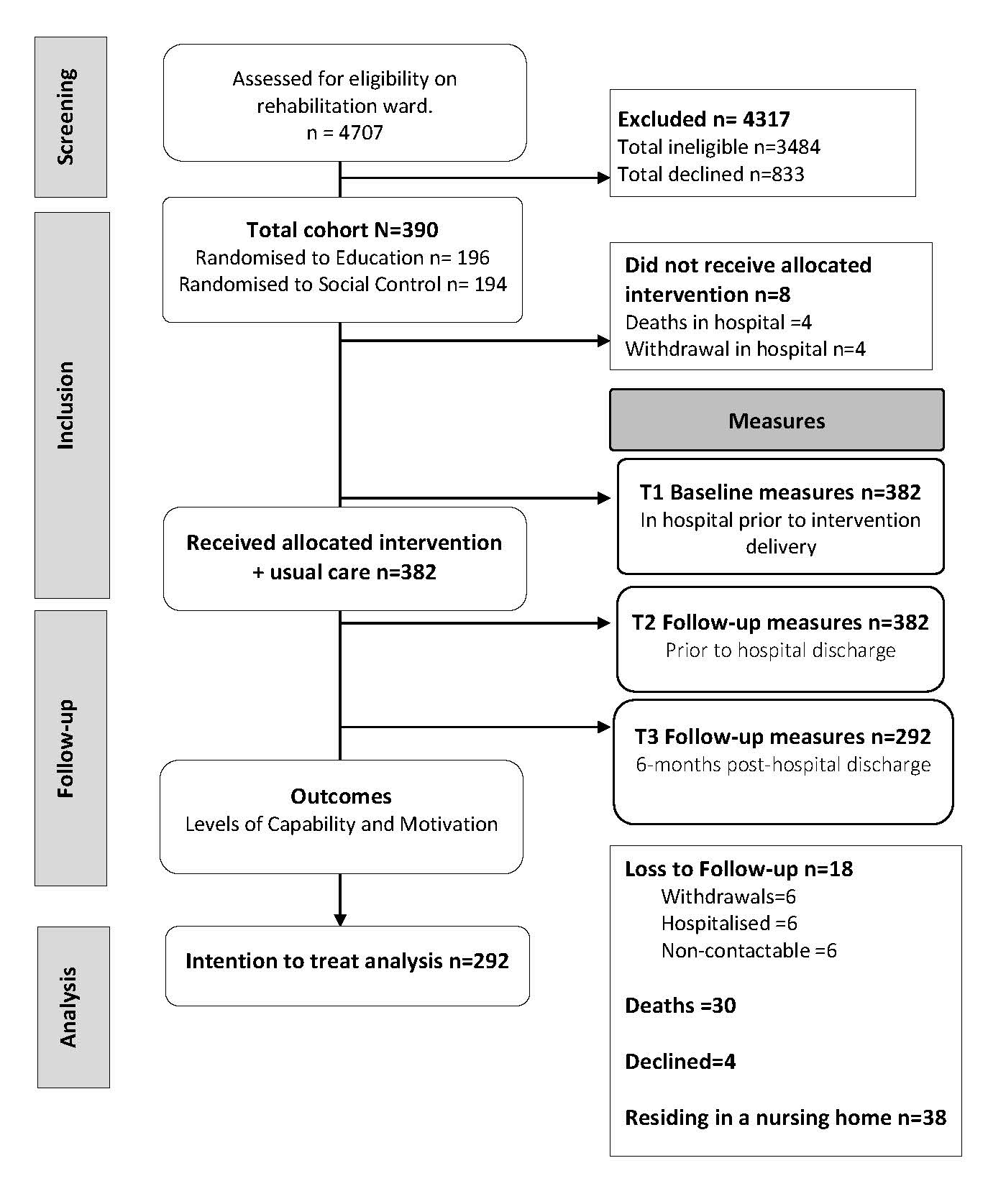

Supplement: Supplementary Figure 1 — Participant flow through the study. [file Image_1.JPEG]
