# Supplementary material for: Tailored Education Increased Capability and Motivation for Fall Prevention in Older People After Hospitalization
Source: Front Public Health. 2021 Aug 3;9:683723. doi: 10.3389/fpubh.2021.683723 (PMC8369365; doi:10.3389/fpubh.2021.683723)
Supplement: Supplementary Table 1 — Descriptive statistics of capability and motivation for education and control groups. [file Data_Sheet_1.pdf]

Supplementary Table 1. Descriptive statistics of capability and motivation for education and control groups

**Outcome: Item**[a]	**Education Group** Median (IQR)			**Control Group** Median (IQR)		
	T1[b] n=195	T2 n=195	T3 n=149	T1 n=187	T2 n=187	T3 n=143
Capability						
1. Knowledge of other older peoples' post-hospitalization falls risk	2 (2-3)	2	2	2 (2-3)	2	2
2. Knowledge of other older people's falls-injury risks following hospitalization [c]	2	2		2(1.5-2)	2	
Motivation						
3. Awareness of own post-hospitalization falls risks	3 (2-4)	2	4	3 (2-4)	3 (2-4)	4
4. Awareness of own post-hospitalization falls-injury risks	2 (2-4)	2	2 (2-3)	2 (2-3)	2 (2-3)	2
5. Awareness of own post-hospitalization loss of independence risks	2 (2-4)	2	1 (1-1)	2 (2-4)	2	1

Notes: **a** item is capability or motivation outcomes measured using median (IQR) of likert scale responses where 1=strongly agree is a better outcome than 2=agree 3=unsure 4=disagree 5=strongly disagree **b** Data collection timeframes: T1in hospital prior to education T2 post-education pre-discharge T3 is 6 months post-discharge **c** this item was omitted in the final survey

Supplementary Table 2. Capability and motivation: complete Likert scale responses for education and control groups at baseline and follow-up

**Outcome:** **Item**[a]		**Education Group** Number (%) Response[b] (T1 n=195; T2 n=195; T3 n=149)					**Control Group** Number (%) Response[b] (T1 n=187; T2 n=187; T3 n=143)				
		SA	A	U	D	SD	SA	A	U	D	SD
Capability:											
1. Knowledge of other older peoples' post-discharge falls risk	T1	34(17)	99(50)	23(11)	31(15)	8(4)	21(11)	109(58)	18(9)	33(17)	6(3)
	T2	84(43)	102(52)	3(1)	0	0	38(20)	111(60)	19(10)	11(6)	3(1)
	T3	74(49)	54(36)	12(8)	8(5)	1	69(48)	55(38)	5(3)	11(7)	3(2)
2. Knowledge of other older peoples' post-discharge falls -injury risk	T1	49(25)	124(63)	7(3)	10(5)	5(2)	46(24)	122(65)	11(6)	8(4)	0
	T2	74(38)	108(55)	5(2)	2(1)	0	40(21)	124(66)	5(2)	10(5)	3(1)
	T3[c]	No data					No data				
Motivation:											
3. Awareness of own post-discharge falls risks	T1	15(7)	73(37)	26(13)	60(30)	21(10)	8(4)	80(42)	10(5)	74(39)	15(8)
	T2	52(26)	107(55)	17(8)	12(6)	1(0.5)	20(10)	70(37)	20(10)	62(33)	10(5)
	T3	5(3)	41(27)	5(3)	56(37)	42(28)	6(4)	34(23)	3(2)	56(39)	44(30)
4. Awareness of own post-discharge falls-injury risks	T1	25(12)	97(49)	15(7)	48(24)	10(5)	28(14)	97(51)	17(9)	42(22)	3(1)
	T2	53(27)	118(60)	7(3)	11(5)	0	20(10)	101(54)	21(11)	36(19)	4(2)
	T3	22(14)	84(56)	15(10)	22(14)	6(4)	32(22)	80(56)	11(7)	15(10)	5(3)
5. Awareness of own post-discharge loss of independence risks	T1	26(13)	99(50)	11(5)	45(23)	14(7)	22(11)	100(53)	6(3)	50(26)	9(4)
	T2	109(55)	70(35)	9(4)	1(0.5)	0	26(14)	98(52)	9(5)	43(23)	6(3)
	T3[c]	100(77)	26(20)	2(1)	1(0.8)	0	96(72)	30(22)	5(3)	1(0.7)	1(0.7)

Notes: **a** item is capability or motivation outcomes measured using number (%) of Likert Scale responses where 1=strongly agree is a better outcome than 2=agree 3=unsure 4=disagree 5=strongly disagree **b** Data collection timeframes: T1in hospital prior to education T2 post-education at discharge T3 is 6months post-discharge **c** At T3 30 participants (n=18 intervention, n=12 control) declined to complete survey item 5 due to individual time limitations
